# Light at the end of the tunnel: FRAP assays combined with super resolution microscopy confirm the presence of a tubular vacuole network in meristematic plant cells

**DOI:** 10.1093/plcell/koae243

**Published:** 2024-09-21

**Authors:** David Scheuring, Elena A Minina, Falco Krueger, Upendo Lupanga, Melanie Krebs, Karin Schumacher

**Affiliations:** Plant Pathology, University of Kaiserslautern-Landau, Paul-Ehrlich Straße 22, 67663 Kaiserslautern, Germany; Department of Molecular Sciences, Uppsala BioCenter, Swedish University of Agricultural Sciences and Linnean Center for Plant Biology, P.O. Box 7015, Uppsala SE-750 07, Sweden; Cell Biology, Centre for Organismal Studies (COS), Heidelberg University, Im Neuenheimer Feld 230, 69120 Heidelberg, Germany; Cell Biology, Centre for Organismal Studies (COS), Heidelberg University, Im Neuenheimer Feld 230, 69120 Heidelberg, Germany; Cell Biology, Centre for Organismal Studies (COS), Heidelberg University, Im Neuenheimer Feld 230, 69120 Heidelberg, Germany; Cell Biology, Centre for Organismal Studies (COS), Heidelberg University, Im Neuenheimer Feld 230, 69120 Heidelberg, Germany; Cell Biology, Centre for Organismal Studies (COS), Heidelberg University, Im Neuenheimer Feld 230, 69120 Heidelberg, Germany

## Abstract

Plant vacuoles play key roles in cellular homeostasis, performing catabolic and storage functions, and regulating pH and ion balance. Despite their essential role, there is still no consensus on how vacuoles are established. A model proposing that the endoplasmic reticulum is the main contributor of membrane for growing vacuoles in meristematic cells has been challenged by a study proposing that plant vacuoles are formed de novo by homotypic fusion of multivesicular bodies (MVBs). Here, we use the *Arabidopsis thaliana* root as a model system to provide a systematic overview of successive vacuole biogenesis stages, starting from the youngest cells proximate to the quiescent center. We combine in vivo high- and super-resolution (STED) microscopy to demonstrate the presence of tubular and connected vacuolar structures in all meristematic cells. Using customized fluorescence recovery after photobleaching (FRAP) assays, we establish different modes of connectivity and demonstrate that thin, tubular vacuoles, as observed in cells near the quiescent center, form an interconnected network. Finally, we argue that a growing body of evidence indicates that vacuolar structures cannot originate from MVBs alone but receive membrane material from different sources simultaneously.

## Introduction

Vacuoles are the largest organelles of plant cells and play a key role in cellular homeostasis, performing catabolic and storage functions, regulating pH and ion balance and, are vital for the adaptability of plant cells to environmental changes (Bassham and Raikhel 1997; Marty 1999; Martinoia et al. 2007; Krüger and Schumacher 2018; Shitan and Yazaki 2020). All plant cells contain vacuoles, however, these organelles are highly dynamic and vary greatly in size, morphology and content, depending on plant species, cell type, developmental stage and environmental conditions (Martinoia et al. 2007; Shitan and Yazaki 2020). Plant vacuoles typically undergo massive structural changes during cell differentiation and growth, starting as relatively small organelles in meristematic cells and taking up to 90% of the cell volume in differentiated cells (Krüger and Schumacher 2018; Kaiser and Scheuring 2020). It is intriguing how such vast reorganization of the organelles can be synchronized with their critical role in cellular homeostasis and responses to environmental stresses. In this context it remains important to determine the source of the vacuolar membrane (tonoplast) that supports the massive reorganization and growth of this organelle (Viotti et al. 2013; Krüger and Schumacher 2018; Cui et al. 2020).

The presence of vacuoles in all plant cells suggests that these organelles are likely to be inherited from cell to cell but does not exclude the possibility that a subpopulation of vacuolar structures might be formed de novo. Understanding the mechanisms of vacuole biogenesis is crucial for elucidating the role of these organelles in plant physiology. However, the pleiotropic effects of mutations affecting vacuolar biogenesis (Isono et al. 2010; Gao et al. 2014; Singh et al. 2014) and the embryonic lethality of mutants with impaired vacuole formation (Rojo et al. 2001) have made it difficult to gain the required knowledge. For these reasons, the more than a century-old debate on the origin of plant vacuoles is still ongoing (Vries 1885; Went 1888).

So far it has been suggested that plant vacuoles might originate from plastid-like structures (Vries 1885), or from Golgi-associated endoplasmic reticulum (ER) that undergoes autophagy-dependent modifications (Marty 1978), or are established with participation of ER-derived provacuoles (Viotti et al. 2013). The most recent model by Cui et al. (2020) proposed a new hypothesis, according to which plant vacuoles start as small vacuoles (SVs) that are de novo produced in young meristematic cells via homotypic fusion of multivesicular bodies (MVBs). However, the available evidence strongly suggests that homotypic fusion of MVBs alone would not produce fully functional vacuolar structures. We review recent work and present additional evidence indicating that vacuolar structures do not originate from MVBs alone but receive membrane material from different sources simultaneously.

### Background on MVBs and vacuole biogenesis

In agreement with existing data, Cui et al. (2019) demonstrated that vacuolar structures and MVBs are decorated with two different sets of membrane proteins. Using immunolabeling, it was shown that YFP-VAMP711 decorated only the tonoplast of young vacuoles, while YFP-Rab5 was restricted to MVBs (Cui et al. 2019). This observation alone suggests that it is unlikely that the formation of the vacuolar membrane is solely dependent on the MVBs. It plausibly requires an additional membrane source that would deliver the characteristic tonoplast transmembrane proteins. Indeed, as we have shown previously, the vacuolar H^+^-ATPase and the vacuolar H^+^-PPase, the two most abundant tonoplast proteins that are already present on tubular vacuolar membranes of meristematic cells, are absent from MVBs (Viotti et al. 2013).

Nevertheless, post-Golgi trafficking in general and MVBs in particular undoubtedly play an important role in plant vacuole establishment. MVBs are essential for the trafficking of proteins from ER and plasma membrane to the vacuole (Cui et al. 2016). During this process, intraluminal vesicles (ILVs) of MVBs accumulate cargo sorted for delivery to the vacuole and are released into the vacuolar lumen upon MVB fusion with the tonoplast (Cui et al. 2016). The presence of ILVs in the vacuolar lumen reported by Cui et al. (2019) is thus a well-described phenotype that merely indicates functioning endomembrane trafficking to the vacuole. The mechanism regulating cargo sorting, formation of ILVs and fusion of MVBs with the tonoplast is relatively well described and known to involve factors implicated in vacuolar maintenance (Isono et al. 2010; Singh et al. 2014; Cui et al. 2016). Consequently, disruption of MVB functionality has a major impact on vacuole morphology (Isono et al. 2010; Singh et al. 2014; Zheng et al. 2014; Kolb et al. 2015). Importantly, cells with aberrant MVB machinery are not devoid of tonoplast membrane material, but typically contain large amounts of it. However, these mutants fail to organize the tonoplast into an organelle with the typical vacuole morphology (Isono et al. 2010; Gao et al. 2014; Zheng et al. 2014). Furthermore, some of the mutants with impaired MVB functionality have highly aberrant vacuole morphologies in young cells, where the network appears to be undergoing intensive fission and fusion events but have relatively normal large lytic vacuoles in differentiated cells (Isono et al. 2010; Zheng et al. 2014). These data suggest that MVBs do not serve as the sole source of the vacuolar membrane, but rather play an important role in balancing factors required for tonoplast organization.

Furthermore, a number of previous studies implementing whole-cell 3D transmission electron microscopy (TEM) (Seguí-Simarro and Staehelin 2006), single-section TEM (Marty 1999) or confocal microscopy (Viotti et al. 2010; Krüger and Schumacher 2018) reported that plant vacuoles at the early stages of their establishment comprise a dynamic tubular network that might undergo temporary fragmentation during cell division. Cui and colleagues suggest that the tubular morphology of young vacuoles observed in some of the previous studies might be an artifact caused by the fluorescent dyes (e.g. 2′,7′-bis-(2-carboxyethyl)-5-(and-6)-carboxyfluorescein [BCECF], SNARF) that have been used to label the vacuolar lumen, as well as a lack of spatial resolution with an unclear developmental context (Viotti et al. 2013). We addressed these points and performed a series of experiments that provide a systematic overview of vacuole formation and allowed us to re-evaluate our previous results.

### Analysis of plant vacuolar morphology during vacuole biogenesis

**Figure 1.**
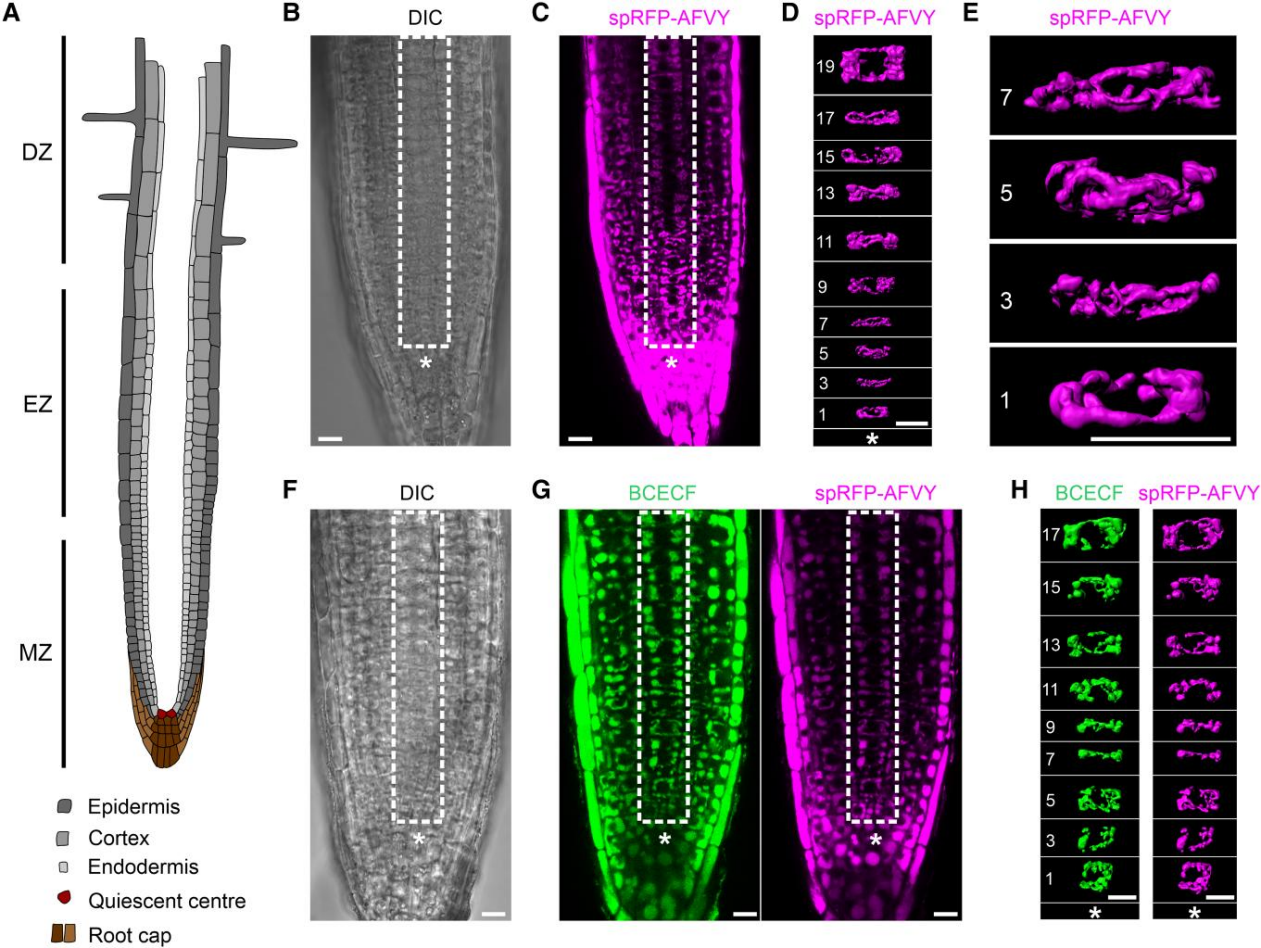
Vacuole morphology at successive stages of biogenesis visualized with the fluorescent dye BCECF and the endogenously expressed vacuolar marker spRFP-AFVY. **A)** Schematic representation of an Arabidopsis root highlighting cell types relevant to this study. The MZ contains the youngest cells with the earliest stages of vacuoles close to the QC. The EZ contains cells that entered the differentiation program resulting in cell elongation along the vertical root axis. The DZ is generally defined by the presence of highly elongated cells and root hairs. **B to H)** To visualize vacuolar lumens in Arabidopsis seedlings expressing the vacuolar marker spRFP-AFVY RFP (magenta) was imaged alone **(B** to **E)** or was co-stained with BCECF (green) **(F** to **H)**. Obtained z-stacks were then processed to generate 3D renderings of vacuoles in cortical cells of a single cell file. Successive stages of vacuole development were traced from the youngest cells closest to the QC. *Note:* staining with BCECF shows no discernible effect on vacuole morphology. Importantly, vacuoles have a distinct tubular morphology even in the youngest cells of the root cortex. **B)** DIC channel showing a single optical slice from a z-stack. **C)** RFP channel of a single optical slice taken from the same z-stack as in **(B)**, to indicate cortex cell file used for rendering in **(D)**. **D)** 3D rendering of the z-stack illustrated in **(C)** showing vacuoles at successive stages of development in a single file of cortex cells. **E)** Enlarged view of **(C)**. **F)** DIC channel showing a single optical slice from a z-stack. **G)** Green (BCECF) and red (spRFP-AFVY) fluorescent channels of a single optical slice from a z-stack to show position of the cortex cells used for rendering in **(G)**. **H)** 3D rendering of the z-stack illustrated in (G) showing vacuoles at successive stages of development in the single file of cortex cells. Asterisks indicate QC. Numbers indicate cell position relative to the QC. White rectangles denote area used for 3D reconstruction of vacuoles in cortex cell files. Scale bars indicate 10 *µ*m.

Here, we used the *Arabidopsis thaliana* (Arabidopsis) primary root as a model, as it is an excellent system for tracking successive stages of cell differentiation and therefore optimal for studying vacuolar development in the meristematic zone (MZ), which is maintained by the quiescent center (QC). Stem cells located in proximity of the QC, give rise to lineages of cell types that later undergo elongation and differentiation (Lee et al. 2013; Fig. 1A). As a result, a single vertical tier of cells represents a snapshot of successive differentiation stages, with the earliest steps occurring close to the QC and the latest occurring proximal to the hypocotyl.

To assess whether BCECF, a dye commonly used to visualize the vacuolar lumen (Scheuring et al. 2015), affects vacuolar morphology, we compared phenotypes of vacuoles in Arabidopsis root cells in the presence of the dye (Fig. 1, B to H) and compared them to vacuole morphologies of cortex cells expressing the well-established vacuolar marker spRFP-AFVY (Hunter et al. 2007). To ensure that our observations are representative, we assessed vacuole morphologies at successive stages of differentiation within the same cell type. For this, we performed high-resolution 3D confocal microscopy of a root area encompassing several cell layers of the meristematic and elongation zones. The imaging data was then used to select a single file of cortex cells, in which cell positions were numbered counting from the QC and 3D reconstructions were made for the vacuoles of every second cell (Fig. 1, B to E, Video 1). We selected cortex cells for analysis, since the 3D TEM reconstructions presented by Cui and authors were predominantly performed on this cell type (Cui et al. 2019).

Consistent with previously published confocal laser scanning microscopy (CLSM) data (Krüger and Schumacher 2018), single section EM (Marty 1999) and whole-cell 3D EM (Seguí-Simarro and Staehelin 2006), we observed a tubular vacuolar network in all meristematic root cells (Fig. 1E) expressing the vacuole marker spRFP-AFVY. The diameter of the tubules was increasing with the age of the cells, eventually causing the network to fuse into more spherical vacuolar structures that occupied most of the volume in the differentiated cells (Fig. 1, B to E; Video 1). Seedlings expressing spRFP-AFVY were then stained with BCECF and imaged as described above. Our data clearly show that BCECF-staining does not induce discernible changes in vacuolar morphology at any of the observed stages of vacuole formation (Fig. 1, F to H). This confirms the validity of the results obtained in previous studies using BCECF as vacuole marker.

To further validate the previous findings on the tubular and network-like structure of vacuoles during the earliest stages of development, we used the recently published RUBY reporter line (He et al. 2020). The synthetic *RUBY* cassette encodes the enzymes required for the synthesis of betalains, tyrosine-derived plant pigments that naturally occur in families of the order *Caryophyllales* (Guerrero-Rubio et al. 2020). In transgenic Arabidopsis lines expressing *RUBY* under control of the *UBQ10* promoter, betalains accumulate exclusively and uniformly inside vacuoles, making them ideal for labeling and studying vacuole morphology (Supplementary Fig. S1A). Spectral analysis of hypocotyl and root tissues revealed the presence of at least two different betalain species, the green-yellow fluorescent betaxanthins and the red fluorescent betacyanins (Supplementary Fig. S1, B to E). Quantification of fluorescence emission showed that hypocotyls accumulated betacyanins to a much greater extent than roots (Supplementary Fig. S1, C and D).

**Figure 2.**
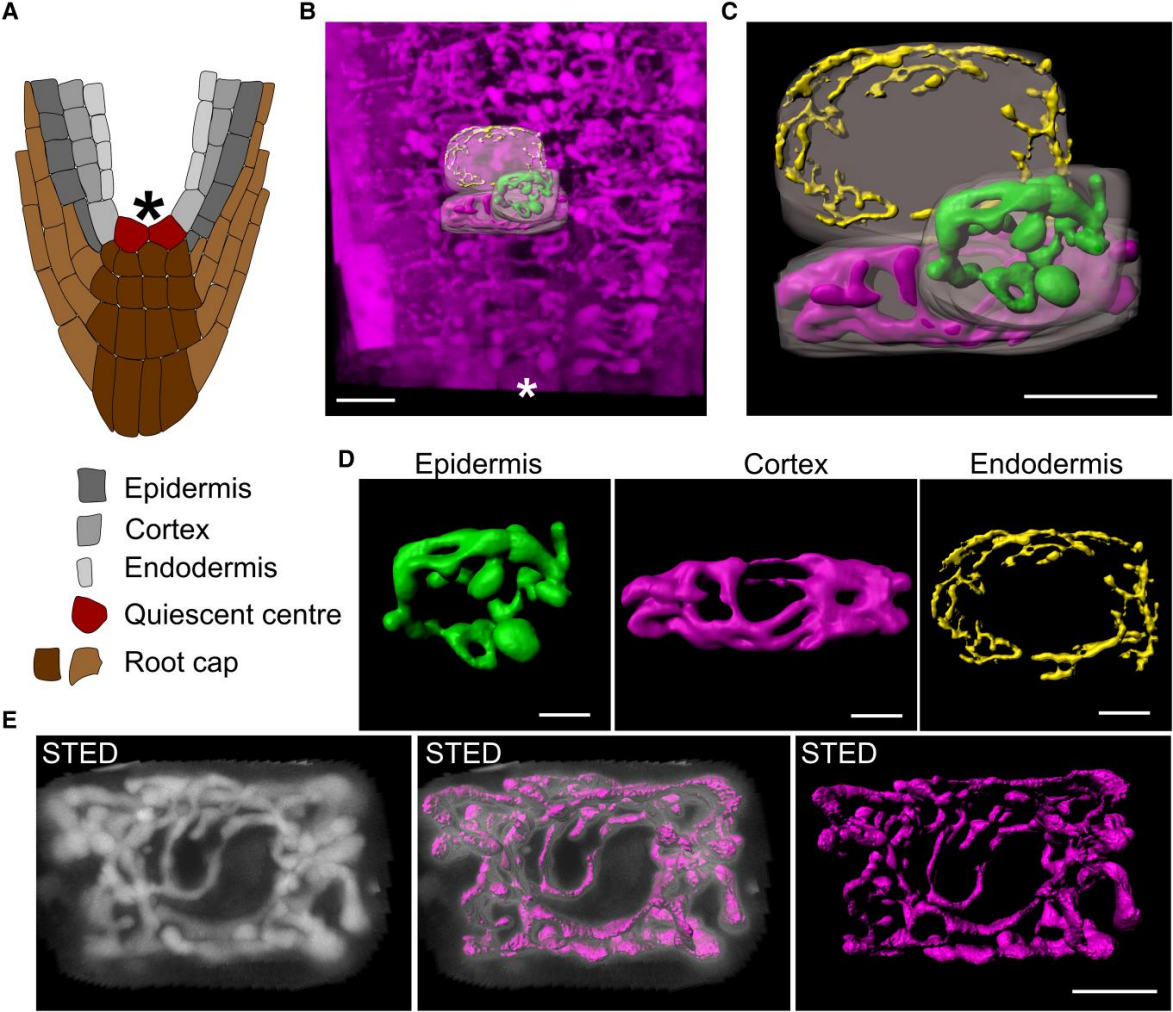
Vacuole morphology in different cell types of the root visualized by betalain fluorescence. **A)** Schematic overview of the root tip close to the QC. **B)** Position of rendered vacuoles in respect to the QC. **C)** 3D vacuole models from epidermal (green), cortex (magenta) and endodermis cells (yellow). Respective cellular volumes are highlighted (transparent white). **D)** Different vacuole morphologies from neighboring cells with different identities. **E)** STED images and 3D model of a vacuole from a cortex cell close to the QC. Scale bars represent 10 *µ*m in **(B)** and **(C)** and 5 *µ*m in **(D)** and **(E)**. Asterisks indicates QC.

Confocal microscopy was performed on roots of seedlings expressing the *RUBY* cassette and z-stacks of cells with different identities located near to the QC were recorded (Fig. 2, A and B). 3D rendered vacuoles from epidermis, cortex, and endodermis cells all showed interconnected tubular structures, with the finest structure in the innermost cell file (Fig. 2, C and D). This confirms our previous findings from cortical cells (Fig. 1) and suggests a network-like structure of vacuoles in young cells, regardless of the cell type. Consistent with this, the use of super-resolution STED (stimulated emission depletion) microscopy allowed us to visualize the tubular vacuole network in living cells with even better spatial resolution at the nanometer scale (Fig. 2E). Since the software for 3D modeling, Imaris (Oxford Instruments), requires significant user input, we display a maximum projection for direct comparison (Fig. 2E). The finest tubular structures we observed were approximately of 300 to 400 nm thickness which is below the described diameter of SVs (400 to 1,000 nm; Cui et al. 2019). However, the resolution, that can be achieved with STED microscopy is still below the spatial resolution of TEM and therefore limits our conclusions to some extent. For example, the identification of ER-derived provacuolar structures (Viotti et al. 2013) remains challenging with light microscopy techniques and has only been visualized using electron microscopy.

**Figure 3.**
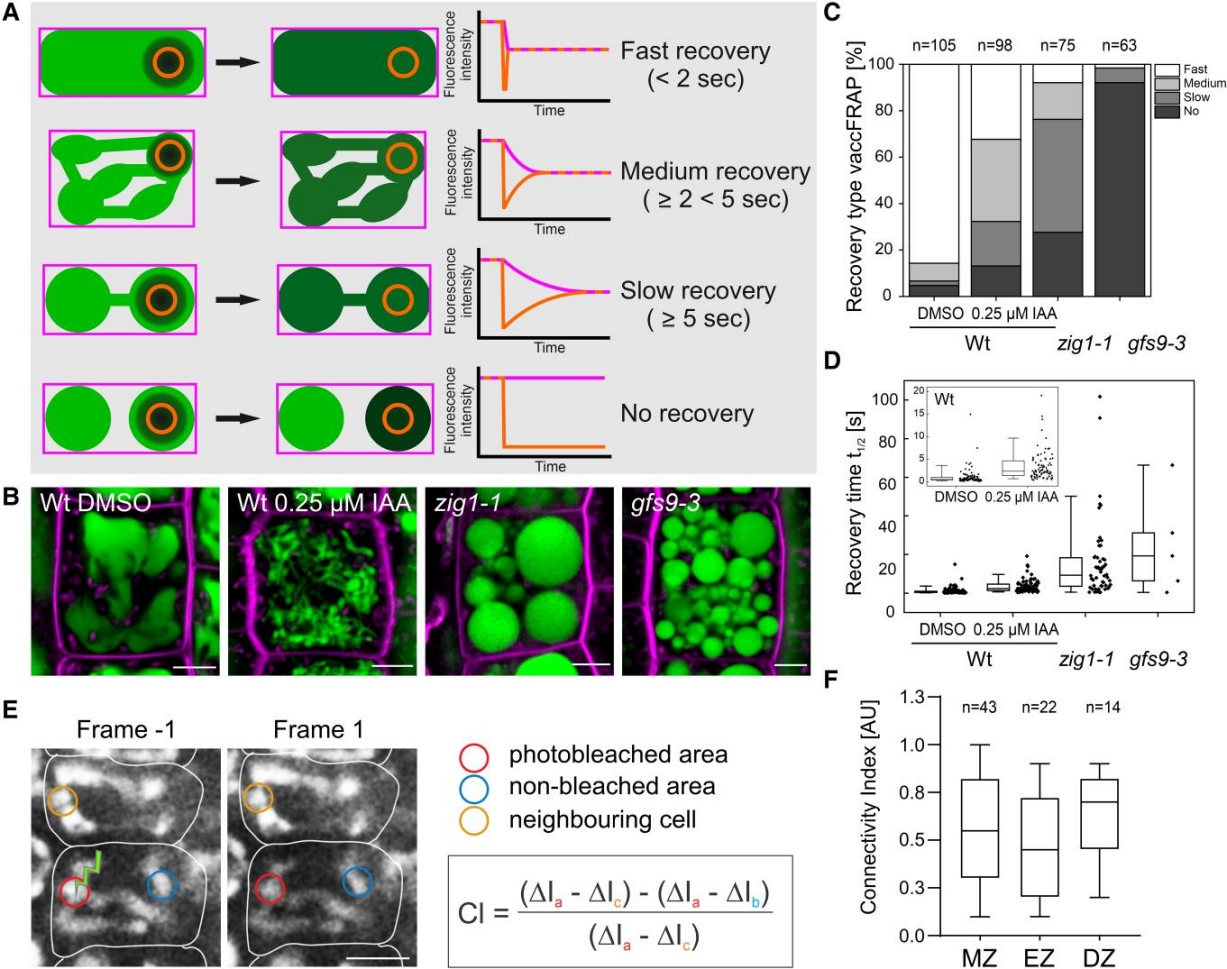
Quantification of vaccFRAP. **A)** Model illustrating the principle of the vaccFRAP`` assay. Colored outlines indicate the areas of fluorescence intensity measurements, with the total vacuole areas labeled in magenta and the bleached areas labeled in beige. Fluorescence intensity behavior of different vacuole connectivity types is indicated by the line graphs. Based on their recovery times, vacuoles were grouped into different connection types ranging from fast, medium, and slow to no recovery. **B)** Representative vacuole phenotypes of BCECF (green) and FM4-64 (magenta) stained seedlings used for vaccFRAP. All measurements were performed in root epidermal cells of 5-d-old BCECF-stained Arabidopsis seedlings. Scale bars represent 5 *μ*m. **C)** Distribution of fluorescence recovery types for different treatments and mutants are given in percent with *n* = 105 for DMSO, *n* = 98 for 0.25 *µ*M IAA, *n* = 75 for *zig1-1*, and *n* = 63 for *gfs9-3*. **D)** Box plot shows fluorescence recovery times (*t*_1/2_) for different treatments and mutants with *n* = 100 for DMSO, *n* = 86 for 0.25 *µ*m IAA, *n* = 54 for *zig1-1*, and *n* = 5 for *gfs9-3*. Inset shows magnification of mock and IAA treated WT samples. Different letters indicate significant differences between samples (Mann–Whitney test, *P*-value < 0.05). **E)** Frames from a FRAP series performed on highly tubulated vacuoles of young cortex cells. “Frame −1”, the frame acquired right before photobleaching. “Frame 1”, the first frame after photobleaching. White lines indicate cell walls. Scale bar: 10 *µ*m. Colored ROIs correspond to colored letters in the equation for the CI calculation. **F)** CI of vacuoles from MZ (cells at the positions 1 to 10, counted from QC), EZ (cells at the positions 14 to 19), and DZ (cells at the positions 20 and upwards). CI = 1 indicates that photobleached and nonphotobleached areas are fully connected and diffusion rate between them is higher than the scanning speed. CI values higher than 0.1 indicate localization of the fluorophores in connected compartments, CI values lower than 0.1, indicate that fluorophores are located in separated compartments. For more information see Materials and methods section. For statistical analysis, a two-tailed Student’s *t*-test was conducted: *P*-value > 0.05. The chart shows representative data of one out of six FRAP experiments, comprised of seven roots to acquire 63 FRAP series.

To assess whether the seemingly connected vacuolar structures indeed represent a shared luminal space, we monitored the diffusion of the fluorescent marker BCECF in the vacuolar lumen using fluorescence recovery after photobleaching (FRAP; Fig. 3). In brief, the fluorophores within a small vacuolar area were exposed to a high intensity laser pulse, resulting in an irreversible loss of fluorescence (photobleaching) at the point of bleaching. The time required to recover fluorescence intensity at the site of bleaching was then measured to assess the extent to which fluorescent dye diffusion is restricted within the vacuolar lumen. As the diffusion time of the dye directly reflects the complexity and connectivity of the vacuolar structures, we termed this method vacuole-connectivity FRAP (vaccFRAP). Similar to our previous work (Scheuring et al. 2016), we classified vacuoles into different categories according to their fluorescence recovery behavior: fast (high connectivity, <2 s), medium (medium connectivity, ≥ 2 < 5 s), slow (low connectivity, ≥5 s) and no recovery (no connectivity; Fig. 3A). To validate the vaccFRAP assay and our classification system, we performed FRAP on vacuoles with significantly different morphologies. Mock-treated wild-type (WT) vacuoles from meristematic cells of the root epidermis were compared with constricted and tubular vacuoles treated with 0.25 *µ*m auxin (indole-3-acetic acid; IAA) and with apparently fragmented vacuoles from *zig1-1* and *gfs9-3* mutants (Yamauchi et al. 1997; Kato et al. 2002; Ichino et al. 2014) (Fig. 3B). The *zig1-1* (*ZIGZAG1*) mutant carries a point mutation in VTI11, a soluble N-ethylmaleimide-sensitive-factor attachment receptor (SNARE) required for membrane trafficking to the vacuole and for homotypic vacuole fusion (Surpin et al. 2003; Zheng et al. 2014) while *GREEN FLUORESCENT SEED 9* (*GSF9*) encodes a *Drosophila melanogaster* ortholog of endosomal maturation defective (ema; Kim et al. 2010, 2012). In Arabidopsis, GFS9 has been shown to contribute to intracellular membrane trafficking and flavonoid accumulation, but its molecular function is unknown (Ichino et al. 2014). Ninety-five percent of all vacuoles measured in mock-treated WT cells had connected volumes, and even for vacuolar structures that appeared disconnected in single optical confocal sections, we observed a rapid recovery of fluorescence (Fig. 3D). Auxin-induced changes of vacuole morphology resulted in a slower recovery, but the overall percentage of connected vacuoles was still 87% (Fig. 3D), indicating more tubular connections by vacuolar constrictions as previously reported (Löfke et al. 2015; Scheuring et al. 2016). Only if vacuolar structures were truly isolated, no fluorescence recovery was observed (Fig. 3D; *zig1-1* and *gfs9-3*). While in case of *zig1-1*, the majority of vacuoles still showed medium and slow fluorescence recovery times, *gfs9-3* vacuoles did not recover their fluorescence, indicating that most of the vacuolar structures are not connected. Taken together, vaccFRAP allows the quantification of even small changes in vacuolar connectivity without the need for 3D microscopy. It is therefore a reliable and elegant method for assessing single cell vacuolar connectivity beyond the optical resolution limitations of light microscopy. Importantly, we demonstrate that most of the highly tubular and filiform vacuolar structures observed after auxin treatment represent an interconnected vacuolar network. Consequently, we infer that the young cells in close proximity to the QC, which also exhibit thin and tubular vacuolar structures, do indeed form an interconnected network.

Although the vaccFRAP assay described above, provided reliable results to discriminate between different vacuolar morphologies in the transition/elongation zone, we realized that performing the assay on plant vacuoles in the meristematic zone was challenging because the vacuolar networks of the youngest meristematic cells are of small size and very dynamic. The small size and rapid movement of the vacuolar structures caused fluorescence intensity fluctuations that interfered with accurate quantification of the intensity changes originating from fluorophore diffusion inside the vacuolar lumen. Therefore, we established a connectivity index (CI) for plant vacuoles (Fig. 3E) as a quantitative measure of the connectivity of the vacuolar network in a cell. To determine the CI in cells of different developmental stages, we used Arabidopsis seedlings expressing the vacuolar marker spL-RFP (Hunter et al. 2007). A CI value of 1.0 indicates unrestricted diffusion of a fluorophore inside the vacuolar lumen. CI values below 0.1 represent a lack of diffusion due to fluorophore localization in separated compartments. We calculated CIs for cells of the meristematic zone (MZ), which contain the youngest vacuoles, the elongation zone (EZ), which contains cells with vacuoles that have undergone substantial fusion, and the differentiation zone (DZ), in which cells contain large, interconnected vacuoles with a balloon-like shape. Statistical analysis revealed no significant difference in the CIs for all three zones (Fig. 3F), confirming that even at the earliest stages of their development, plant vacuoles comprise a dynamic network of interconnected tubules.

## Conclusions

Notably, Cui and colleagues observed fragmentation of vacuoles using super-resolution microscopy on fixed cells in experiments involving photoconversion of a vacuole marker in living cells (Cui et al. 2019). Interestingly, their photoconversion experiments revealed fragmented vacuoles in highly elongated cells (ratio cell length to width greater than 2:1), which should typically contain connected vacuoles in WT plants under normal growth conditions. It is well established that plant vacuoles can undergo drastic morphological changes, including fragmentation, in response to environmental stimuli (Bassham and Raikhel 1997; Gao et al. 2005; Krüger and Schumacher 2018; Shitan and Yazaki 2020; Cao et al. 2022). Recently, tonoplast-localized mechanosensory proteins in tip-growing cells of *Physcomitrium patens* were shown to directly affect vacuole morphology (Radin et al. 2021), confirming that differences in mechanical stimulation due to sample handling could potentially lead to structural changes. We speculated that the discrepancies between our observations and the data reported by Cui and colleagues might originate from differences in plant growth conditions and different sample treatments. In particular, Cui et al. (2019) cut Arabidopsis roots as a prerequisite for high-pressure freezing, whereas live cell imaging does not require seedlings to be damaged. It cannot be excluded that mechanical stress and damage, which may occur during sample preparation could induce fragmentation of tubular vacuoles. To test the robustness of the vacuole phenotypes observed in our studies, we performed a comparative analysis of vacuolar morphology of seedlings grown in two independent laboratories located in two different countries. We reproducibly observed tubular vacuolar networks in young cells of seedlings grown, stained, and imaged at different facilities (Supplementary Fig. S2). Additionally, we compared vacuole morphologies of seedlings collected directly from the growth plate with vacuoles from seedlings incubated in liquid medium for 3 and 72 h to simulate different sample handling commonly used for staining and induction conditions. Both, maximum projections and reconstructed 3D vacuoles from all conditions displayed tubular connections between larger vacuolar substructures (Supplementary Fig. S3). Therefore, we are confident that under standard growth conditions and even after prolonged incubation in liquid medium, young plant vacuoles typically consist of an interconnected tubular network. Our results provide systematic evidence that validates and confirms previous observations (Marty 1999; Seguí-Simarro and Staehelin 2006; Viotti et al. 2013; Krüger and Schumacher 2018). Undoubtedly, MVB fusion occurs, and the resulting SVs described by Cui et al. contribute to vacuole biogenesis. However, the fact that even the youngest meristematic cells contain tubular vacuoles that are already acidified by vacuolar proton pumps (Viotti et al. 2013) cannot be neglected, and therefore the two proposed mechanisms are not mutually exclusive as both types of early vacuolar structures have been reported (Cui et al. 2019).

Taken together, in this study we argue that (I) the existing knowledge on plant endomembrane trafficking does not support the hypothesis of vacuolar structures resulting from homotypic fusion of MVBs alone. (II) Using a combination of 3D reconstructions and STED, we provide a comprehensive overview of plant vacuolar morphology at successive stages of their development, starting from the youngest cells proximal to the QC, and show that even at the earliest stages, vacuoles have a tubular network morphology. Furthermore, (III) we find that BCECF does not affect vacuolar morphology, confirming conclusions from previous studies using this dye, and (IV) we introduce the RUBY reporter to visualize vacuoles in deeper tissue layers via betalain fluorescence. Finally, (V) we use a customized FRAP assay (vaccFRAP) to show that thin and highly tubular vacuoles as observed at the earliest stages of development, consist of an interconnected network.

## Materials and methods

### Plant material and growth

In this study we used *A. thaliana* Col-0 accession WT plants and transgenic lines expressing the spRFP-AFVY, spL-RFP (Hunter et al. 2007) or UBQ:RUBY (He et al. 2020) reporter, respectively. Seeds were surface sterilized for 20 min in 70% ethanol with 0.05% Triton X-100, washed in 95% ethanol and air dried. Seedlings were grown on vertical plates with 0.5 × MS (M0222, Duchefa), supplemented with 10 mm MES (M1503, Duchefa), 1% sucrose, and 0.8% plant agar (P1001, Duchefa), pH5.8 at 22°C under long-day conditions (150 *µ*m light, 16 h light, 8 h dark).

### BCECF staining

BCECF Acetoxymethyl Ester (B1150 ThermoFisher) was stored as a 10 mm stock solution in DMSO. For staining, 5 to 7 d-old seedlings were submerged in liquid medium with 0.5 × MS (M0222, Duchefa), 10 mm MES (M1503, Duchefa), 1% sucrose (pH 5.8) containing 10 *µ*m BCECF and incubated at room temperature for 2 h, followed by washing for 15 min in fresh 0.5 × MS liquid medium and immediate mounting for imaging.

### Confocal microscopy

Images for 3D-reconstructions of vacuoles were obtained using a Leica SP5, a Leica SP8 or a Zeiss LSM880 (INST 248/254-1) confocal microscope. Drift corrections of z-stacks were carried out using the FIJI plugin “StackReg”. For imaging using the Leica SP8 CLSM a 63 × objective (NA 1.30) was used, for imaging with the Zeiss LSM880, AxioObserver SP7 a C-Apochromat 40× (1.2 W AutoCorr M27) objective was used. With the Leica systems, BCECF was excited at 488 nm and its emission was detected at 490 to 552 nm, while RFP fluorescence was excited at 561 nm and its emission was detected at 577 to 700 nm, both using the standard mode of the HyD detectors. Pinhole was set to 1 AU, scanning speed was set to 400 Hz, line average to 3, pixel resolution was set to optimal, and number of optical slices was system optimized. The used settings for the Zeiss LSM880, AxioObserver SP7 were similar. Betalains were imaged with 489 nm excitation and 500 to 700 nm emission. Experiments were performed at COS, Heidelberg University, and University of Kaiserslautern-Landau, Germany. Additionally, observed vacuolar phenotypes were tested on seedlings grown at Uppsala BioCenter, SLU, Sweden using a Zeiss LSM 800 microscope equipped with GaAsp detectors and ZEN 2 software, 63× water immersion (NA 1.20) objective. Excitation and emission settings were set similarly to the ones described above.

### 3D reconstruction

For 3D reconstructions of vacuoles Imaris 9.2 (Oxford Instruments) was used. For each reconstructed cell, cell walls were manually traced using at least every third slice of the z-stack. The tracing data was then used to create a cell surface reconstruction. In the next step, the surface of the analyzed cells was used to create a masked channel, in which signal intensity for the voxels outside the selected cell surface were set to 0. This channel was then employed to automatically create a 3D model of vacuolar lumen of the analyzed cell. The obtained model was visually compared and adjusted to the underlying signal.

### Super-resolution (STED) microscopy

A Leica—Stellaris 8 (INST 248/293-1 FUGG) equipped with a white light laser and a 775 nm STED laser was used. For image acquisition, a HC PL APO CS2 93× glycerin immersion objective, NA 1.3 was used and unidirectional scan mode with 600 Hz and 5% MotCorrPosition was employed to acquire z-stacks. Betalains were excited with 560 nm light and emission detected from 570 to 750 nm. STED laser intensity was 100% in the tauSTED modus.

### Vacuole-connectivity fluorescence recovery after photobleaching

Vacuole connectivity measurements were done in epidermal cells of 5-d-old seedling root tips that had been stained with BCECF as described above. FRAP experiments were set up using the *FRAP Wizard* included in the Leica LASAF software. Imaging settings in all measurements were identical. Images of 512 × 512 pixels were acquired with a scan speed of 1,400 Hz (bi-directional mode) at a zoom-factor of 6 and 3-times line-averaging. Hybrid detectors were operated in standard detection mode. Altogether the settings resulted in a temporal resolution of 575 ms. Cells containing vacuoles of interest were randomly selected and the focal plane was adjusted to the equatorial plane of the cell. For bleaching the *Point-Bleach* mode was used. The target area, a vacuole sector that appeared strongly convoluted or isolated, was selected setting a single bleach-point for 300 to 500 ms. The bleach laser power was adjusted between 35% and 50%. For the FRAP experiments, four pre-bleach frames, one single bleach frame, and 50 to 200 post-bleach frames were recorded. A maximum of five FRAP measurements per root were conducted. Recovery times, *t*_1/2_ were calculated from time series by selecting single exponential recovery as curve fitting method using the Fiji plugin *FRAP Profiler* (Lynch et al. 2012). FRAP measurements with a mobile phase fraction below 30% were considered to not recover their fluorescence and their recovery times were not included to quantify fluorescence recovery times (Fig. 3D). Vacuoles with recovery times ≥5 s were grouped as slow recovering, vacuoles with recovery times ≥ 2 < 5 s were grouped as medium speed recovering vacuoles, and vacuoles with recovery times smaller than 2 s were considered to be fast recovering vacuoles (Fig. 3C). Significant differences of recovery half-times between samples were determined using Mann–Whitney test with a *P*-values < 0.05.

### Connectivity index

High motility of young vacuoles in meristematic cells was causing significant fluctuations of fluorescence intensity within a selected area, masking intensity changes originating from fluorophore diffusion. Thus, the standard FRAP approach could not be efficiently applied to vacuoles within meristematic cells close to the QC. Hence, we further adapted the vaccFRAP approach described above. We ratio that diffusion of the fluorophore within the aqueous environment of the vacuole is a rapid unrestricted process and photobleaching of a small area would lead to a detectable drop of fluorescence in the whole vacuolar network. To assess the rapid changes in the fluorophore distribution we analyzed FRAP series frames right before (Frame −1) and right after (Frame 1) photobleaching, measuring changes in the fluorescence intensities within selected areas. Within these frames, we selected a small vacuolar area that was exposed to high intensity laser (area “a” within the red circle in Supplementary Fig. S4) and an area of the vacuolar network most distant from the photobleached region (area “b” within the green circle in Supplementary Fig. S4). Furthermore, an area in the vacuolar network of neighboring cell (area “c” within the blue circle in Supplementary Fig. S4) was used as a control, since its vacuole was definitely not connected to the vacuolar network of the assessed cell.

CI was then calculated in the following steps:

- Δ*I*_a_ = (*I*_a(Frame 1)_ − *I*_a(Frame −1)_) * 100/*I*_a(Frame 1)_; intensity drop in the photobleached area of the vacuole as % of this area intensity before photobleaching.
- Δ*I*_b_ = (*I*_b(Frame 1)_ − *I*_b__(Frame −1)_) * 100/*I*_b(Frame 1)_; intensity drop in the not photobleached area of the same vacuole as % of this area intensity before photobleaching.
- Δ*I*_c_ = (*I*_c(Frame 1)_ − *I*_c(Frame −1)_) * 100/*I*_a(Frame 1)_; intensity drop in the vacuole of neighboring cell as % of this area intensity before photobleaching.
- Rel = Δ*I*_a_ − Δ*I*_b_; relative loss of fluorescence within the vacuole, comparing photobleached and nonphotobleached areas (areas are in potentially connected parts of vacuole).
- Ref = Δ*I*_a_ − Δ*I*_c_; reference loss of fluorescence, comparing photobleached area in one cell and nonphotobleached in another (areas are in disconnected vacuoles).
- CI = (Ref − Rel)/Ref; Connectivity index.

CI = 0 indicates that photobleached and not photobleached areas are not connected. CI = 1 indicates that photobleached and nonphotobleached areas are fully connected and diffusion rate between them is higher than the scanning speed. Cut-off value, the lowest CI value that corresponds to connected network was estimated empirically. To establish the cut-off value for this study we used cells of the differentiation zone as the positive control representing connected vacuoles and vacuoles in neighboring cells as a negative control, representing disconnected vacuolar structures. Under our conditions, the CI values lower than 0.1 reproducibly correlated with nonconnected vacuolar compartments. Please note, that CI < 0 means that nonphotobleached area lost more signal than the photobleached area, indicating technical issues most probably resulting from sample drift during scanning. Similarly, CI > 1 indicates that there is a technical issue, most probably sample drift during scanning.

CIs were quantified using the designated semiautomated assay implementing ImageJ macros (https://github.com/AlyonaMinina/Connectivity-Index). The macros were designed to first process Leica project files and extract individual FRAP series as tiff stack files and then guide the user for selecting the areas required for quantification of the CI index.

## Supplementary data

The following materials are available in the online version of this article.

**Supplementary Figure S1.** Betalains accumulate uniformly and exclusively in vacuoles of seedlings expressing the RUBY cassette.

**Supplementary Figure S2.** Tubular vacuoles are reproducibly detected in young cortical cells of seedlings grown at two independent research facilities.

**Supplementary Figure S3.** Vacuolar morphology upon incubation in liquid medium.

**Supplementary Figure S4.** CI allows quantitative comparison of fluorescence recovery in highly mobile vacuolar structures of young root cells.

## Supplementary Material

koae243_Supplementary_Data

## Data Availability

The data underlying this article will be shared on reasonable request to the corresponding author.

## Dive Curated Terms

The following phenotypic, genotypic, and functional terms are of significance to the work described in this paper:

UBQ10 Gramene: AT4G05320
UBQ10 Araport: AT4G05320
