# Supplementary material for: Light at the end of the tunnel: FRAP assays combined with super resolution microscopy confirm the presence of a tubular vacuole network in meristematic plant cells
Source: Plant Cell. 2024 Sep 21;36(11):4683–91. doi: 10.1093/plcell/koae243 (PMC11635288; doi:10.1093/plcell/koae243)
Supplement: koae243_Supplementary_Data [file koae243_Supplementary_Data.pdf]

Scheuring et al. Light at the end of the tunnel: FRAP assays combined with super resolution microscopy confirm the presence of a tubular vacuole network in meristematic plant cells. Plant Cell. 2024.
Supplementary Figures.

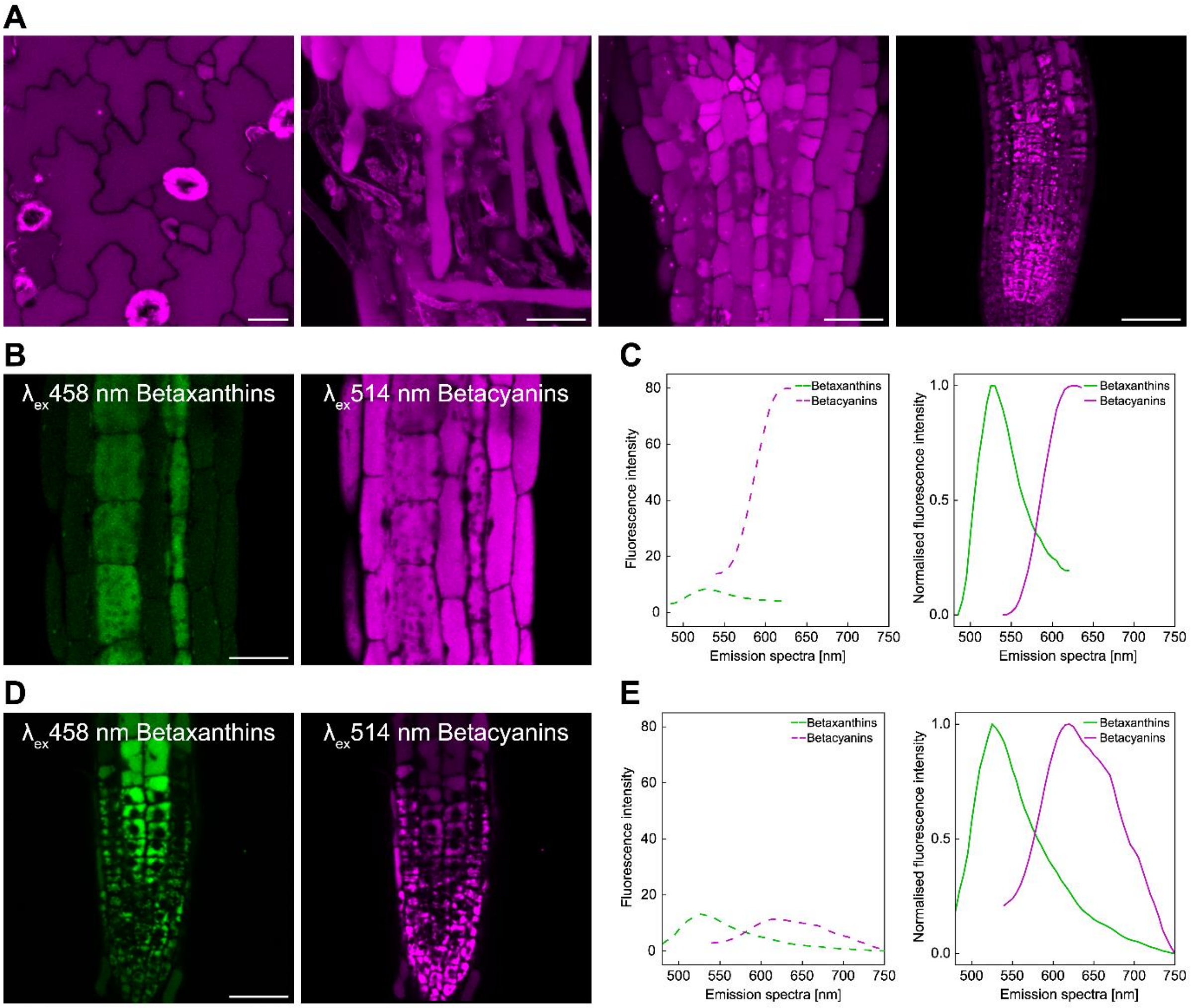

Supplementary Figure S1. Betalains accumulate uniformly and exclusively in vacuoles of seedlings expressing the *RUBY* cassette. **A)** CLSM of betalain-containing vacuoles in cells of the leaf epidermis, the shoot-root transition, the upper hypocotyl and the root tip (left to right). Maximum projections of z-stacks are shown. **B-D)** At least two different species of betalains, the green-yellow fluorescent betaxanthins and the red fluorescent betacyanins, are found in vacuoles of hypocotyl (B, C) and root (D, E) cells. **B, C)** Emission scans of hypocotyl cells. Betaxanthins were excited at 458 nm and emission was recorded from 480-620 nm. Betacyanins were excited at 514 nm and emission was recorded from 540-635 nm. **D, E)** Emission scans from root cells. Betaxanthins were excited at 458 nm and emission was recorded from 480-750 nm. Betacyanins were excited at 514 nm and emission was recorded from 540-750 nm. **C, E)** Fluorescence intensities (left) and normalized fluorescence intensities (right) are shown. Non-normalized fluorescence intensities indicate that hypocotyl cells accumulate more betacyanins than roots. Scale bars represent 50 µm.

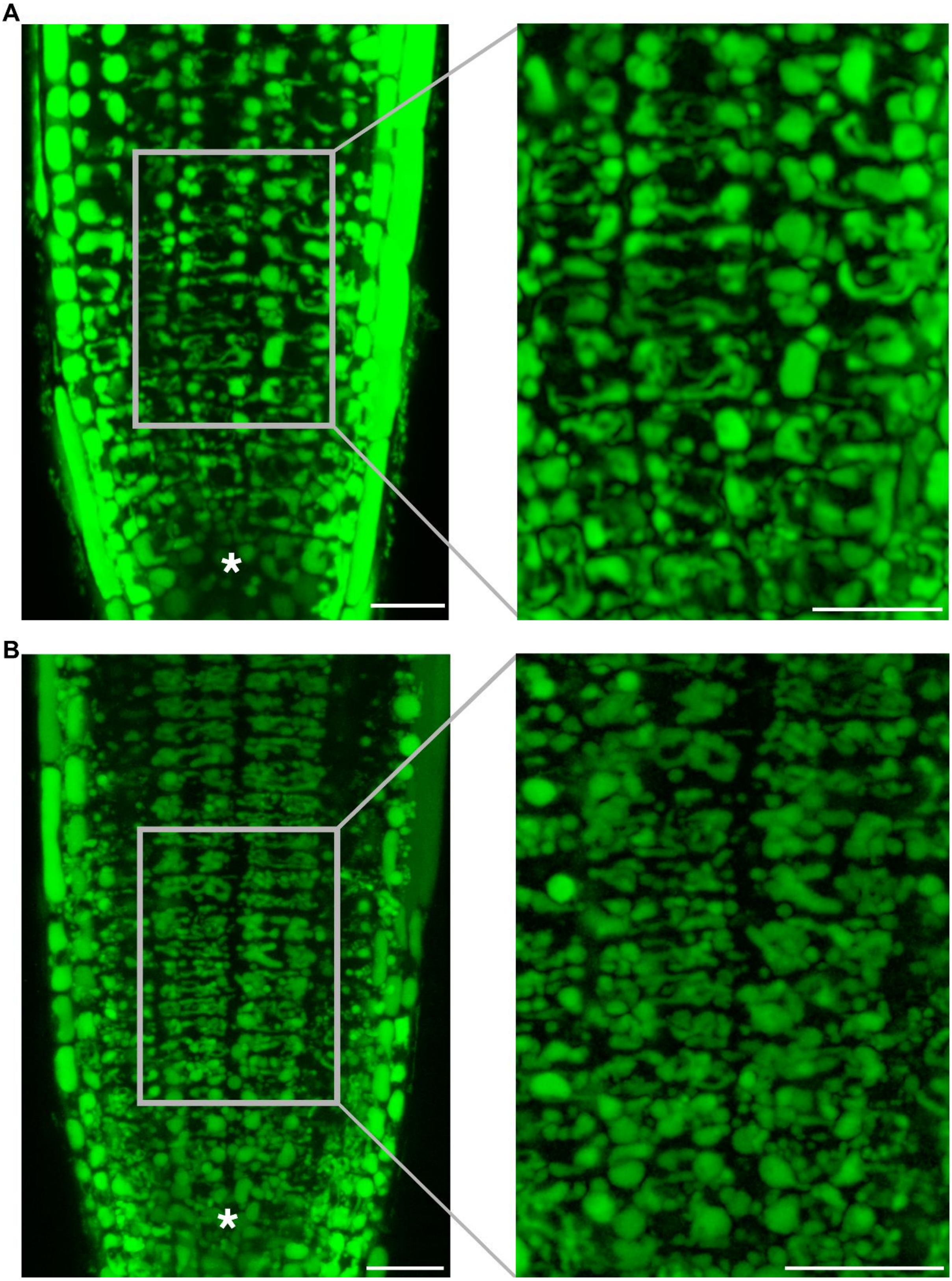

Supplementary Figure S2. Tubular vacuoles are reproducibly detected in young cortical cells of seedlings grown at two independent research facilities. **A)** The spRFP-AFVY transgenic line was grown at COS plant growth facility, University of Heidelberg, Germany. Five-day-old seedlings were stained with BCECF and imaged using a Leica SP8 confocal microscope. The maximum projection of z-stacked BCECF-stained vacuoles shows an intricate tubular vacuolar network in young cells proximal to the QC. **B)** Col-0 wild-type seedlings were grown in the plant facility at Uppsala BioCenter, SLU, Sweden. Staining and imaging were performed as in (A), but using a Zeiss LSM 800 confocal microscope. Asterisks indicate QC. Scale bars represent 20 µm.

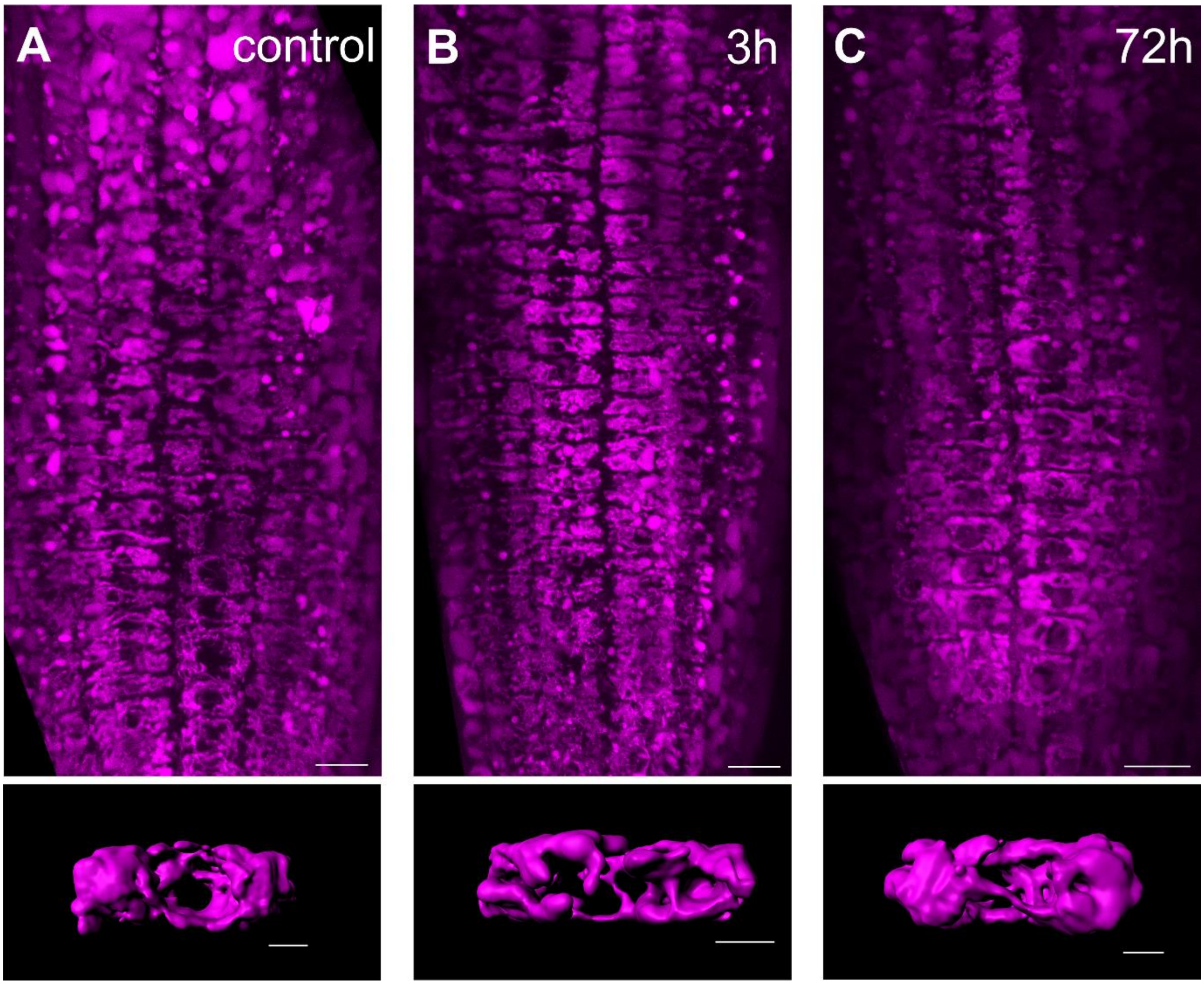

Supplementary Figure S3. Vacuolar morphology upon incubation in liquid medium. The transgenic RUBY line was used to highlight vacuoles in the Arabidopsis root. Maximum projections are displayed and vacuoles from cells close to the QC were reconstructed. **A)** Vacuoles from seedlings directly collected from plate. **B)** 3 h incubation in liquid 0.5 x MS medium. **C)** 72 h incubation in liquid 0.5 x MS medium. Scale bars represent 20 µm (maximum projections) and 5 µm (3D vacuoles).

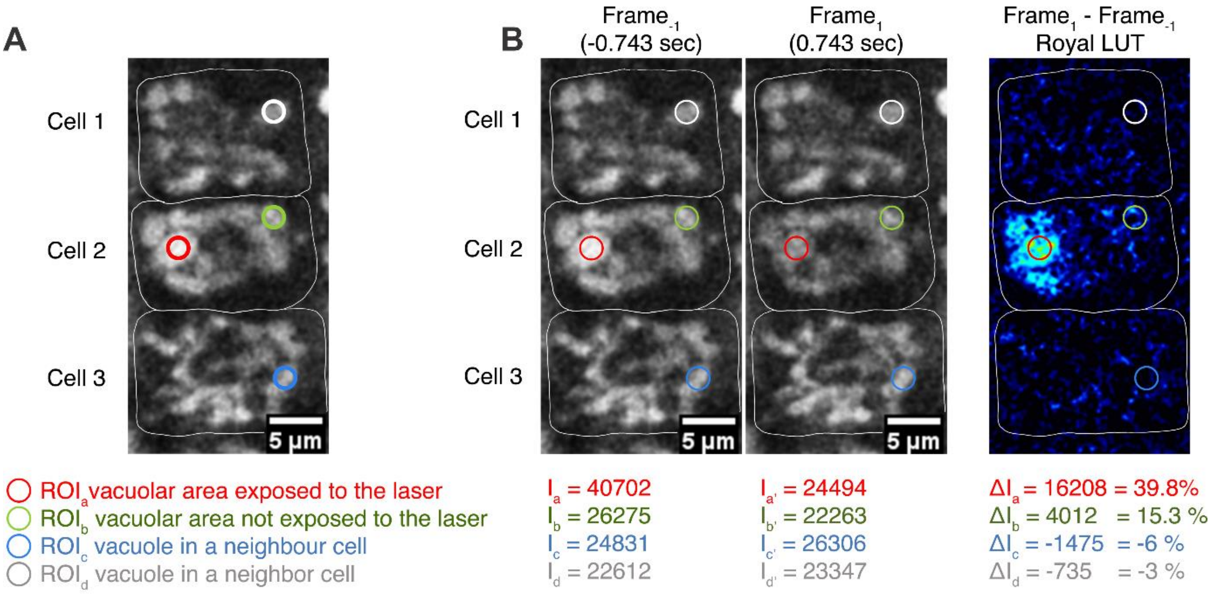

C

$$CI = \frac{(\Delta I_a - \Delta I_d) - (\Delta I_a - \Delta I_b)}{(\Delta I_a - \Delta I_d)} = 0.43$$

$$CI_{negative\ control} = \frac{(\Delta I_a - \Delta I_d) - (\Delta I_a - \Delta I_c)}{(\Delta I_a - \Delta I_d)} = -0.06$$

Supplementary Figure S4. Connectivity Index allows quantitative comparison of fluorescence recovery in highly mobile vacuolar structures of young root cells. **A)** FRAP assay was performed according to the description provided in the Materials and methods chapter using two independent transgenic Arabidopsis lines expressing red fluorescent markers for the vacuolar lumen. An example of regions or interest (ROIs) selected on 3 cells, among which only the ROI1 in cell 2 was exposed to high intensity laser. White lines indicate cell walls. **B)** Frame-1 was taken immediately before the start of photobleaching. Frame1 was acquired right after photobleaching was finished. The numbers signify fluorescence intensity values within the regions of interest measured by using the ImageJ software. To highlight intensity changes caused by photobleaching, Frame 1 image was subtracted from Frame-1 image using the ImageJ "Calculator" function. The resulting image was color-coded using Royal LUT, lighter colors indicate a bigger difference in the intensity. Numbers show that the fluorescence intensity decrease within the ROIs, which are presented as percent of the fluorescence signal within the corresponding ROI before photobleaching. **C)** Connectivity Index (CI) is calculated by comparing change in fluorescence in ROIs within the same cell and in neighboring cells. Comparison of ROIs within the same cell reveals diffusion rate of the fluorophore within vacuolar structures of the cell. Comparison of ROIs located in different cells informs about fluctuations in fluorescence intensity caused by scanning and movement of the vacuoles. CI values higher than 0.1 indicate that the fluorophore can diffuse between vacuolar structures. CI values lower than 0.1 indicate that the fluorophore is trapped in separated structures.
